# Identification and Functional Characterization of PtoMYB055 Involved in the Regulation of the Lignin Biosynthesis Pathway in *Populus tomentosa*

**DOI:** 10.3390/ijms21144857

**Published:** 2020-07-09

**Authors:** Yiming Sun, Sha Ren, Shenglong Ye, Qiaoyan Tian, Keming Luo

**Affiliations:** Chongqing Key Laboratory of Plant Resource Conservation and Germplasm Innovation, School of Life Sciences, Southwest University, Chongqing 400715, China; s8355@swu.edu.cn (Y.S.); liujunchen@email.swu.edu.cn (S.R.); wszzx759684@email.swu.edu.cn (S.Y.); zxc123456789@email.swu.edu.cn (Q.T.)

**Keywords:** *Populus tomentosa*, secondary cell wall, lignin biosynthesis, activator, ectopic deposition

## Abstract

Wood, which is mainly composed of lignified secondary cell wall, is the most abundant biomass in woody plants. Previous studies have revealed that R2R3-type MYB transcription factors are important regulators of the formation of the secondary cell wall in vascular plants. In this study, we isolated the R2R3-type MYB transcription factor gene *PtoMYB055*, which is mainly expressed in xylem and phloem tissue, from *Populus tomentosa* and demonstrate that PtoMYB055 is a key regulator of lignin biosynthesis. PtoMYB055 as a transcriptional activator is localized to the nucleus. Overexpression of *PtoMYB055* upregulates expression of lignin biosynthetic genes in transgenic poplar plants, resulting in ectopic deposition of lignin in phloem tissue and an increase in thickness of the secondary cell wall. In sum, PtoMYB055 is a transcriptional activator that is involved in regulating lignin biosynthesis during the formation of the secondary cell wall in poplar.

## 1. Introduction

Wood, also called secondary xylem, is the most abundant biomass in woody plants. It is widely used in pulp and papermaking and as a potential feedstock in the production of biofuels as alternatives to fossil fuels. Lignin, one of the main components of the secondary cell wall, is made up of polymerized aromatic alcohol. It can be divided into three groups according to its monomers: p-hydroxyphenyl H, guaiacyl G, and syringyl S phenylpropanoid lignin [1,2]. Lignin, one of the most important factors determining the properties of wood, can enhance the tensile stress of three-dimensional crystal fiber, allowing the plant to grow taller and protecting it from invasion by pathogens [3]. In addition, it strongly limits the conversion of biomass to pulp or biofuel [4].

In recent decades, understanding of lignin biosynthesis has increased. More than 10 enzymes, including 4CL (4-coumarate: CoA ligase), C4H (cinnamate-4-hydroxylase), CCR (cinnamoyl-CoA reductase), CAD (cinnamyl-alcohol dehydrogenase), F5H (coniferaldehyde 5-hydroxylase), and CCoAOMT (caffeoyl-coenzyme A 3-O-methyltransferase), are involved in the biosynthesis of lignin [5]. The functions of these enzymes in lignin biosynthesis have been characterized. For example, F5H (ferulic acid 5-hydroxylase), an enzyme in the phenylpropanoid pathway, is one of the control points for S-lignin synthesis [6,7,8]; CCR, as the first step of the redox reaction of lignin biosynthesis, is a limiting enzyme that controls carbon entering the lignin biosynthesis pathway [9,10].

Lignin biosynthesis is a highly regulated process that is controlled at the transcription level. In the complex regulatory network in the modern herbaceous plant *Arabidopsis thaliana*, NAC (NAM, ATAF1/2, and CUC2) and MYB transcription factors act as first- and second-level master switches to regulate a series of downstream transcription factors and genes in biosynthesis of the secondary cell wall [11,12]. MYB is one of the largest families of transcription factors in the plant kingdom; they play important roles in the growth and development of plants and in response to biotic and abiotic stresses [13,14,15,16]. R2R3-type MYB transcription factors are involved in regulating lignin biosynthesis [11,17,18,19]. For instance, AmMYB308 and AmMYB330 from Antirrhinum regulate the biosynthesis of lignin and phenylpropanoid [13]. In many plants, MYB46 and MYB83 act as switches regulating downstream transcription factors in lignin biosynthesis [20,21,22]. Most MYB transcription factors (e.g., MYB58 and MYB63) positively regulate lignin biosynthesis [23], but others (e.g., MYB4, MYB52, and MYB75) play negative roles [24,25,26].

*Populus tomentosa*, a species native to China with high economic and ecological value [27,28], is a good model woody plant for studying wood formation [29]. Previously, we isolated and characterized MYB transcription factors, including PtoMYB74 and PtoMYB92, from *P. tomentosa*. Both of them positively regulate expression of lignin biosynthetic genes, leading to an increase in the thickness of the secondary cell wall [30,31]. In this study, we isolated and functionally characterized the R2R3 MYB gene *PtoMYB055* from *P. tomentosa*. Phylogenetic analyses showed that PtoMYB055 is a homolog of *Arabidopsis* MYB61. Overexpression of *PtoMYB055* in *P. tomentosa* promoted expression of lignin biosynthetic genes, resulting in an increase in the thickness of the secondary cell wall in vessels of transgenic plants. Our findings indicate that PtoMYB055 is a trans-activator that is involved in regulating lignin biosynthesis during the formation of the secondary cell wall in poplar.

## 2. Results

### 2.1. Isolation and Characterization of PtoMYB055 from P. tomentosa

**Figure 1:**
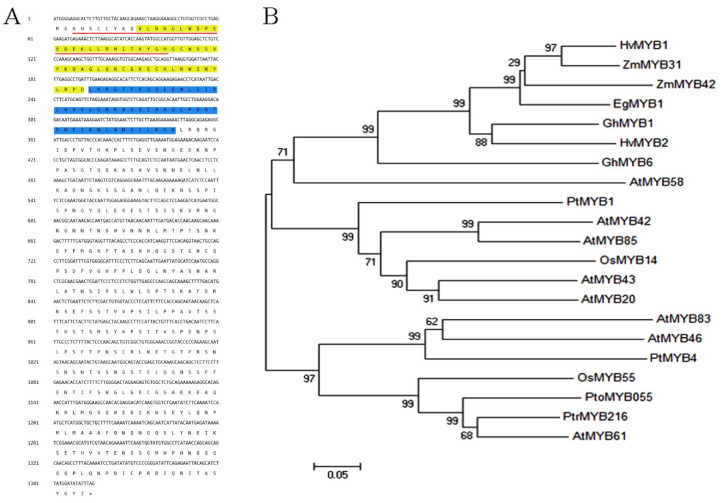
Characterization of the PtoMYB055 protein. (**A**) Amino acid sequence of the coding region of PtoMYB055. The nuclear localization signal is underlined with red line. The R2 MYB domain is highlighted with yellow and the R3 MYB domain was highlighted with blue. (**B**) The phylogenetic relationship between PtoMYB055 and other MYB transcription factors associated with the secondary cell wall was constructed with the neighbor-joining method in MEGA (version 5.1) [35]. Bootstrap values are shown as percentages at the nodes. The GenBank accession numbers of sequences used are given in Section 4.

After isolating *PtoMYB055* cDNA by RT-PCR using gene-specific primers, we aligned PtoMYB055 with its homologs. As shown in Figure 1A, PtoMYB055 contained two highly conserved adjacent R2R3 repeats and a nuclear localization signal (the red line in Figure 1) in the N-terminal region.

A number of R2R3-type MYB transcription factors are involved in regulating the formation of the secondary cell wall in plants [17,18,19,32]. Phylogenetic analysis revealed that PtoMYB055 was clustered with PtrMYB216, AtMYB61, and OsMYB55, which all perform this function (Figure 1B) [18,33,34]. These results suggest that PtoMYB055 is a potential transcriptional regulator of the formation of the secondary cell wall in poplar.

### 2.2. Expression Profiles of PtoMYB055 in Poplar

**Figure 2:**
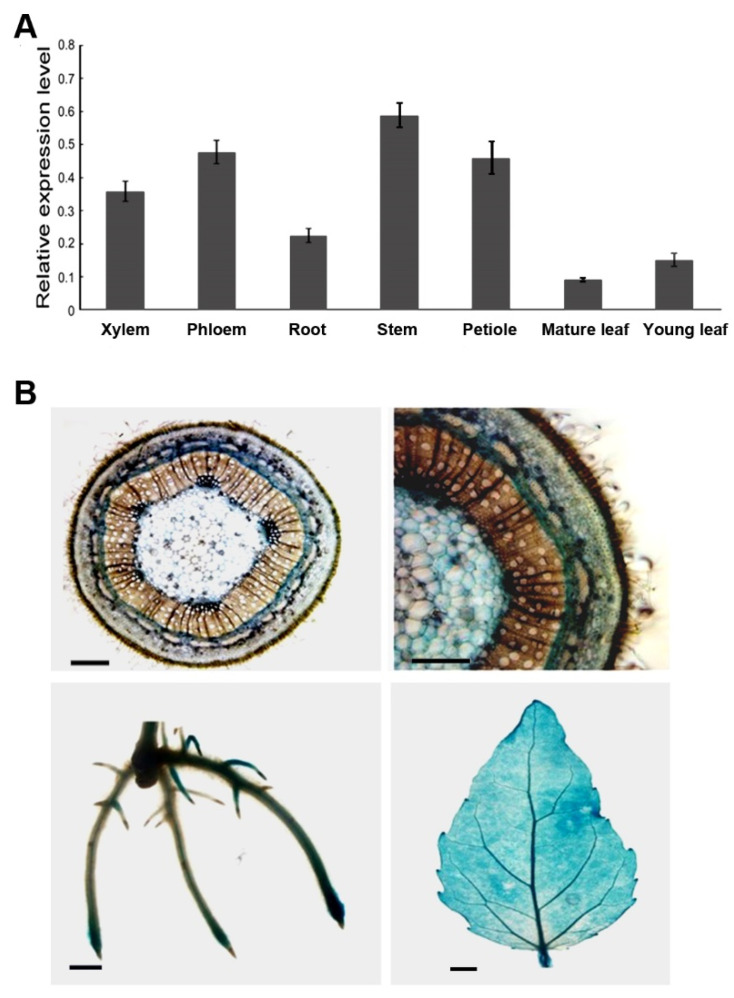
Expression patterns of *PtoMYB055* in poplar. (**A**) Real-time quantitative PCR analysis of *PtoMYB055* transcript levels in various tissues of *Populus tomentosa* Carr. Expression of the *18S* gene was used as a control. Error bars represent ± SD of three biological replicates. (**B**) Promoter analyses of *PtrMYB055* with GUS (*β*-glucuronidase) staining in various tissues of mature *PtoMYB055*:GUS plants. Upper plane, stem, scale bar = 500 µm; lower left plane: root, scale bar = 5 mm; lower right plane: leaf, scale bar = 2 mm.

To determine the expression profiles of *PtoMYB055*, we used quantitative reverse transcription polymerase chain reaction (RT-qPCR) to detect expression of *PtoMYB055* in different tissues of *P*. *tomentosa*. The highest expression occurred in lignin-abundant tissue, such as the stem, xylem, and phloem, but it was also expressed in other tissue (Figure 2A). This suggests that PtoMYB055 might be related to lignin synthesis.

To further investigate spatial and temporal expression of *PtoMYB055*, we constructed a binary vector that contained the GUS (*β*-glucuronidase) controlled by the *PtoMYB055* promoter and transformed it into *Populus tomentosa*. Histochemical GUS staining showed that expression of GUS in different tissues was consistent with the results of RT-qPCR. As shown in the upper panel of Figure 2B, specific expression of the *PtoMYB055* promoter was observed in the protoxylem and phloem of the stem of transgenic *P*. *tomentosa* (Figure 2B). In the root, expression of the *PtoMYB055* promoter was observed in the tips and secondary roots (Figure 2B). However, the *PtoMYB055* expression was detected in all cell types in the leaf and vein. These results indicate that *PtoMYB055* is expressed preferentially in tissue undergoing thickening of the secondary cell wall.

### 2.3. PtoMYB055 is Located in the Nucleus and Acts as a Transcriptional Activator

**Figure 3:**
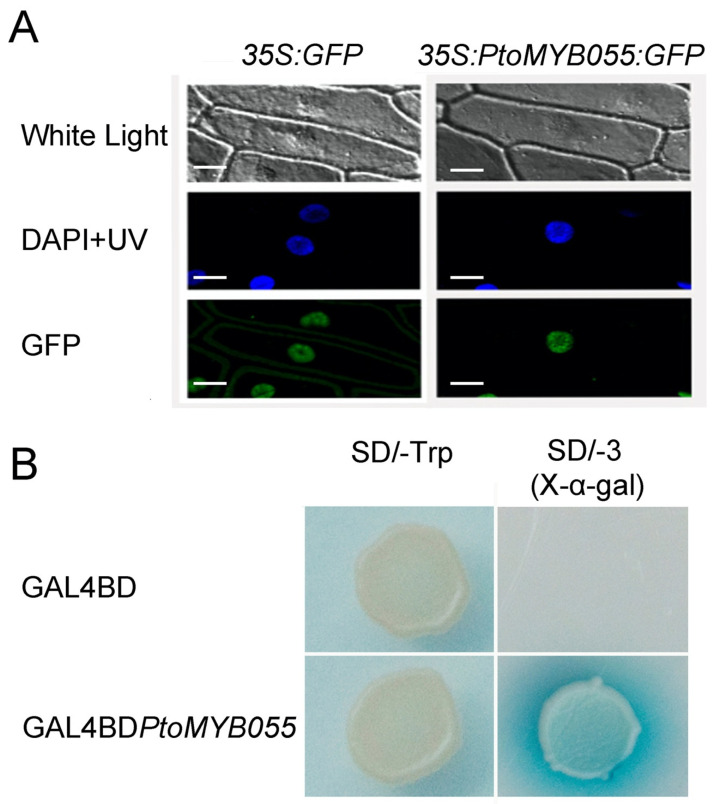
Nuclear localization and transcription activity. (**A**) 35S:Pto-MYB216:GFP and 35S:GFP transformed to onion epidermis. GFP fluorescence was observed with a confocal microscope 18 h after bombardment. The position of the nucleus was confirmed with DAPI staining and compared to a bright field, scale bar = 50 µm. (**B**) PtoMYB055 has trans-activation activity in yeast. On a medium without histidine, adenine, and tryptophan, yeast containing recombinant plasmid, GAL4BD*PtoMYB055*, appeared blue. This shows that PtoMYB055 induce the activity of X-b-gal.

As PtoMYB055 has a nuclear localization signal, we suspected that it may be localized to the nucleus. To confirm this conjecture, we constructed a plasmid in which the coding sequence of *PtoMYB055* and *green fluorescent protein* (*GFP*) was fused and under the control of Cauliflower mosaic virus (CaMV, Caulimovirus family) 35S. We then transformed this plasmid into onion epidermal cells. A robust fluorescent signal was observed in the nucleus, which indicated that PtoMYB055 was located in the nucleus (Figure 3A), consistent with its function as a transcription factor.

To examine whether PtoMYB055 is a transcriptional activator, we fused the CDS of *PtoMYB055* with the *GAL4* DNA-binding domain for transactivation analyses in yeast. We found that yeast harboring GAL4BD-PtoMYB055 can grow normally in a medium lacking leucine, histidine, and adenine. This demonstrates that PtoMYB055 is a transcriptional activator (Figure 3B).

### 2.4. Overexpression of PtoMYB055 in P. tomentosa Increases Lignin Content

**Figure 4:**
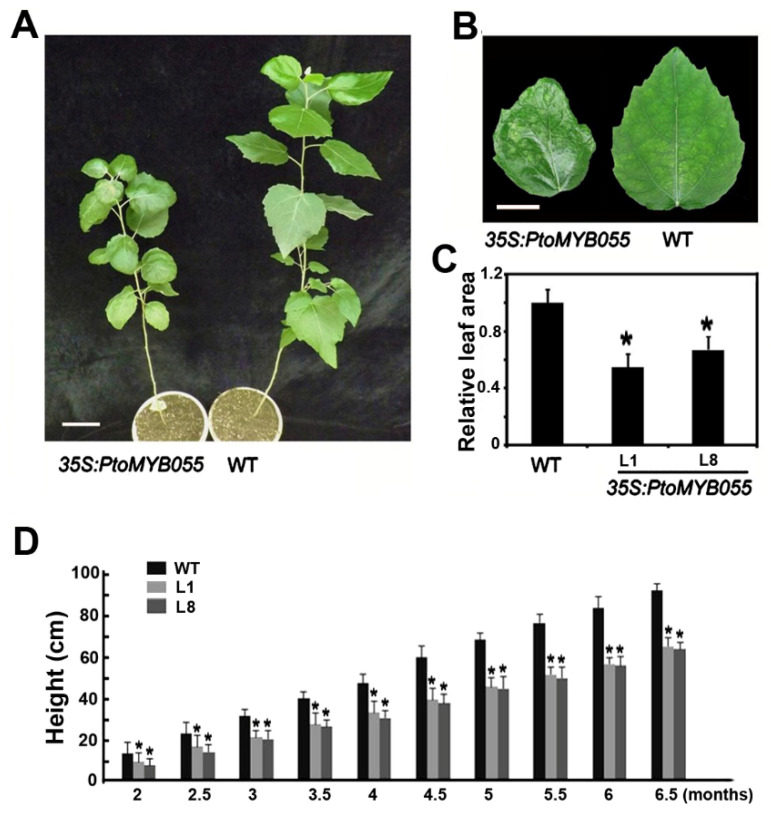
Phenotypes of transgenic poplar plants overexpressing *PtoMYB055*. (**A**) Growth in the 35S-PtoMYB055-overexpressing poplar and the wild type. Scale bar = 10 cm. (**B**) Leaf morphology of the 35S-PtoMYB055-overexpressing poplar and the wild type. Scale bar = 5 cm. (**C**) Relative leaf area of 35S-PtoMYB055 and the wild type. * *p* < 0.05 (*t* test). (**D**) Heights of 35S-PtoMYB055-overexpressing poplar and the wild type over time. * *p* < 0.05 (*t* test).

To detect whether PtoMYB055 is involved in regulating the biosynthesis of the secondary cell wall in *P*. *tomentosa*, we constructed an overexpression plasmid in which *PtoMYB055* was driven by CaMV 35S promoter and transformed it into *P*. *tomentosa* using *Agrobacterium tumefaciens*. Two positive transgenic plants (line 1 and line 8) determined by PCR assay were chosen for further analysis. The results showed some morphological differences between transgenic 35S-PtoMYB055 plants and the wild-type control. Overexpression of *PtoMYB055* dramatically inhibited growth in *P*. *tomentosa*. The height of 35S-PtoMYB055 plants was decreased significantly, by about 33% (Figure 4A), and their leaf area reduced by 40–60% (Figure 4B) compared to the wild type. Moreover, the leaves of 35S-PtoMYB055 plants were curlier than those of the wild type (Figure 4C).

**Figure 5:**
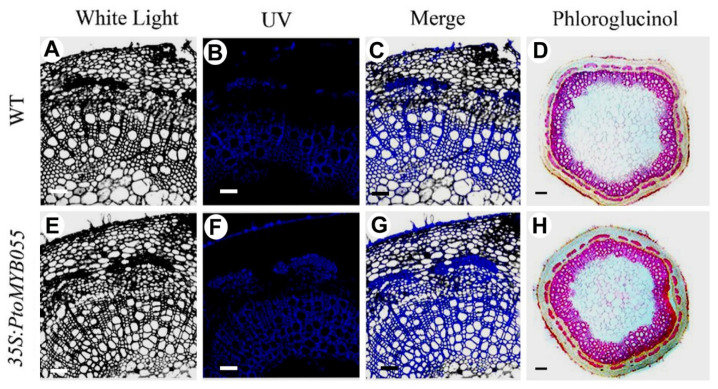
Ectopic deposition of lignin in *PtoMYB055*-overexpressing poplar. (**A**–**C**,**E**–**G**) Laser scanning confocal microscopy of stem sections at the fifth internode in *PtoMYB055*-overexpressing transgenic poplar. (**D**,**H**) Stem sections at the fifth internode were stained by phloroglucinol-HCl. Scale bars = 50 µm (**A**–**C**,**E**–**G**), 500 µm (**D**,**H**).

To test whether lignin deposition in xylem is changed in 35S-PtoMYB055 transgenic plants, we stained the stem of wild-type and 35S-PtoMYB055 transgenic plants with phloroglucinol-hydrochloric acid (HCl). Compared to the wild type, the thickness of the stained area in 35S-PtoMYB055 stems was significantly increased, which indicates that overexpression of *PtoMYB055* led to the ectopic deposition of lignin (Figure 5G,H). Lignin autofluorescence under UV light showed that the thickness of the secondary cell wall of vessels and xylary fibers in 35S-PtoMYB055 plants was dramatically increased compared to the wild type. Furthermore, epidermal cells of 35S-PtoMYB055 leaves exhibited stronger lignin autofluorescence signals than the wild type. However, 35S-PtoMYB055 plants showed ectopic lignin deposition in the stem (Figure 5A–F). In addition, the lignin content of 35S-PtoMYB055 plants increased by 10.27–23.39% compared to the wild type (Table 1). These results demonstrate that overexpression of PtoMYB055 leads to an increase in lignin content in the xylem and ectopic deposition of lignin in epidermal cells.

### 2.5. Overexpression of PtoMYB055 Induces Gene Expression Associated with Biosynthesis of the Secondary Cell Wall in Poplar

**Figure 6:**
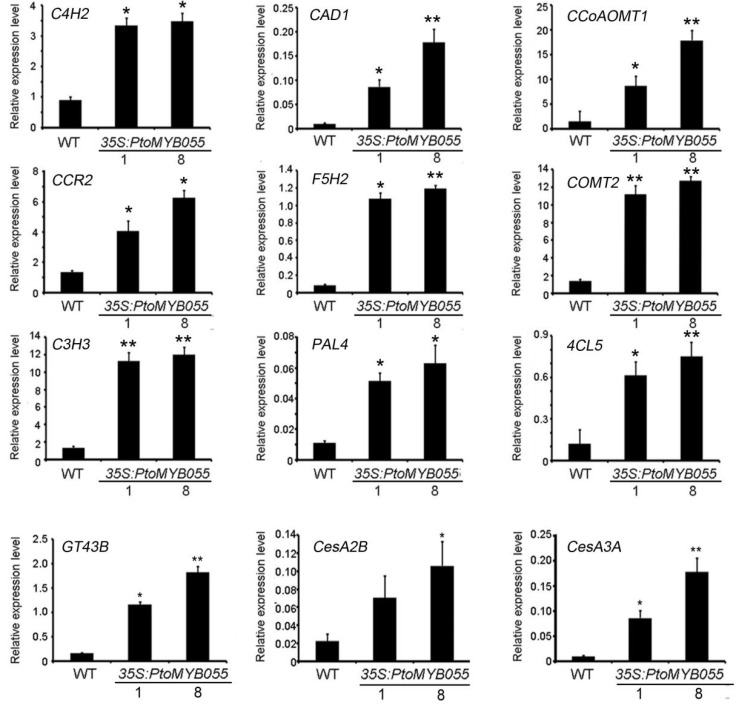
Gene expression analyses of secondary cell wall biosynthetic genes in 35S-PtoMYB055 poplar plants. Transcriptional expression of genes involved in lignin biosynthesis (*C4H4*, *CAD1*, *CCoAOMT1*, *CCR2*, *F5H2*, *COMT2*, *C3H3*, *PAL4*, *4CL5*), xylan (*GT43B*), and cellulose (*CesA2B*, *CesA3A*) was upregulated. The poplar Actin gene was used as an internal control. Error bars represent ± SD of three biological replicates. * *p* < 0.05. ** *p* < 0.01.

To further prove that PtoMYB055 positively regulates the biosynthesis of lignin, we used real-time quantitative PCR to detect relative expression of genes involved in phenylpropanoid metabolism generally and monolignol biosynthesis specifically. The results showed that *PAL4*, *C4H2*, *4CL5*, *C3H3*, *CCOAOMT1*, *CCR2*, *F5H2*, and *COMT2* were significantly upregulated in all 35S-PtoMYB055 plants compared to wild-type plants. In addition, *GT43B*, *CesA2B*, and *CesA3A*, which are involved in xylan and cellulose biosynthesis, were also upregulated (Figure 6). These data suggest that PtoMYB055 is indeed involved in regulating the formation of the secondary cell wall.

## 3. Discussion

Lignin is one of the main components of the secondary cell wall [2], which allows plants to grow upward and provides hydrophilicity for the vascular system [4]. In addition, it is a renewable biofuel. In the past few decades, numerous studies have elucidated the lignin biosynthetic pathway, and many enzymes have been isolated and identified [4].

In recent years, interest in the regulation of lignin biosynthesis has increased. MYB, WRKY, NAC, and LIM transcription factors are all involved in regulating lignin biosynthesis [25,36,37]. As one of the largest families of transcription factors in the plant kingdom, MYB transcription factors are involved in regulating lignin biosynthesis. Some (e.g., MYB46 and MYB83) are involved as activators [12,20,22], whereas others (e.g., MYB4 [24], MYB52 [21], and MYB75 [26]) negatively regulate lignin biosynthesis as repressors. Here we found that a MYB transcription factor, PtoMYB055, acts as a transcriptional activator to positively regulate lignin biosynthesis in *Populus tomentosa*. The N terminus of PtoMYB055 contains an R2R3 repeat domain, which is conserved in AtMYB61 and OsMYB55 (Figure 1A). Both OsMYB55 and AtMYB61 act as activators in regulating lignin synthesis during the formation of the secondary cell wall [33,38]. Therefore, we supposed that PtoMYB055 might possess a similar function in poplar. The tissue expression profile revealed that *PtoMYB055* is preferentially expressed in organs in which lignin is abundant, such as xylem and phloem (Figure 2). Overexpression of *PtoMYB055* leads to ectopic deposition of lignin in the secondary cell wall and an increase in lignin content (Figure 5 and Table 1). A yeast activation assay showed that PtoMYB055 is a transcriptional activator (Figure 3). PtoMYB055 might transactivate lignin biosynthetic genes and consequently affect the lignin content. The fact that relative expression of lignin biosynthetic genes is upregulated in *PtoMYB055*-overexpressing lines compared to the wild type confirmed our hypothesis (Figure 6). These results indicate that PtoMYB055 indeed participates in the formation of wood in poplar as a positive regulator. In addition, Li et al. reported that PdOLP1, an Osmotin-like protein in poplar, is a negative regulator of secondary wall biosynthesis and PtoBLH8 and PtoWRKY40 repress the transcriptional activation of its promoter [39]. PtoMYB055 also might regulated the secondary cell wall biosynthesis via activating or repressing other regulators, such as PdOLP1.

MYB transcription factors, one of the largest families of transcription factors in the plant kingdom, are involved in almost all biological processes. Thus, many have multiple functions. For instance, besides regulating the formation of the secondary cell wall, AtMYB61, the PtoMYB055 homolog in *Arabidopsis*, is also involved in stomatal aperture and seed coat mucilage deposition [40,41]. Therefore, we speculate that PtoMYB055 has a similar function in poplar; this hypothesis must be verified in future studies. In the present study, overexpression of *PtoMYB055* decreased the height of poplar and made its leaves curl, findings that have not been reported for overexpression of AtMYB61 in *Arabidopsis* and rice. More interesting is that Romano et al. observed that leaf size increased when AtMYB61 was overexpressed in *Arabidopsis*. This indicates that although the regulation network is conserved among species, members have species-specific functions.

In conclusion, our study characterized a new R2R3-type MYB transcription factor that participates as a transcriptional activator in regulating lignin biosynthesis in poplar. This study will be useful for designing strategies for genetic improvement of wood biomass.

## 4. Materials and Methods

### 4.1. Plant Materials

*P. tomentosa* Carr. (clone 741) was grown in a greenhouse at 25 °C under a 16/8 h light/dark cycle with 5000 lux of supplementary light and relative humidity around 60% for optimal growth [42]. *A. thaliana* (ecotype Columbia) plants were grown in a greenhouse at 22–23 °C under a 16/8 h light/dark cycle with 10,000 lux of supplementary light and humidity of approximately 80%.

### 4.2. Cloning of PtoMYB055

The cDNA fragment encoding *PtoMYB055* was amplified with gene-specific primers (forward: 5′-CATTGCAGCTCAGTCTTTCT-3′, reverse: 5′-CTCCCTGTACCAATGCAAGCT-3′) based on PtrMYB055 (XM_002320893) from *P. trichocarpa* by PCR. The PCR reaction was performed with *Pfu* DNA polymerase (Takara, Dalian, China) in a total volume of 25 µL at 94 °C for 5 min; 32 cycles of 94 °C for 50 s, 58 °C for 50 s, and 72 °C for 100 s; followed by a final extension of 72 °C for 10 min. The PCR product that was sequenced was cloned into the plant binary vector pCXSN [43]. We transformed the resulting vector p35S:PtoMYB055, with the PtoMYB055 open reading frame (ORF) driven by the CaMV 35S promoter, into *A. tumefaciens* strain EHA105 using the freeze-thaw method.

### 4.3. Sequence Comparisons and Phylogenetic Analysis

Database searches of the nucleotide and deduced amino acid sequences were performed through NCBI/GenBank/Blast for sequence alignment [44]. Phylogenic analyses were performed with MEGA (version 5.1).

### 4.4. RT-qPCR Analysis

Total RNA was extracted from roots, stems, young leaves, mature leaves, phloem, xylem, and petioles of poplar plants with TRIzol reagent. Then, genomic DNA was removed from the total RNA, and cDNA was synthesized with a PrimeScript™ RT Reagent Kit with gDNA Eraser (Takara, Dalian, China). We performed RT-qPCR with a TP700 Real-Time PCR Machine (Takara, Dalian, China) using the SYBR Green PCR Master Mix Kit (Takara, Dalian, China) following the manual’s recommendations. The housekeeping *18S* gene was used as a reference gene. Gene-specific primers used in RT-qPCR assays are listed in Table S1.

### 4.5. Subcellular Localization

The ORF of *PtoMYB055* was amplified with gene-specific primers (Table S1) and cloned into the pCX-DG vector [43] to generate a 35S-PtoMYB055:GFP fusion vector, which was induced into onion epidermal cells with Gene Gun (Scientz, Hangzhou, China). The onion skin was stained with DAPI and observed under a confocal microscope (Leica, Wetzlar, Germany).

### 4.6. Transcriptional Activation Assay

The CDS of *PtoMYB055* was cloned into pGBKT7 (Clontech, Mountain View, CA, USA) and introduced into the yeast strain *Saccharomyces cerevisiae* Gold2 as described previously [45]. Transformants were grown on SD medium lacking tryptophan for the selection of a positive clone and then on SD medium lacking tryptophan, histidine, and adenine for the transactivation assay. X-a-gal was used to identify the transcriptional activation activity of PtoMYB055.

### 4.7. Transformation of P. tomentosa Carr.

Poplar transformation was mediated by *Agrobacterium* as described previously [46]. First, leaf disks were infected with *Agrobacterium* cultures containing p35S-PtoMYB055 for 8–10 min. They were subsequently transferred to WPM medium (2.0 mg/L zeatin, 1.0 mg/L 1-naphthalene acetic acid (NAA)). Second, the infected disks were put into co-medium (containing 1 mg/L acetosyringone) in the dark for 2 days and then transferred to callus-inducing medium (containing 2.0 mg/L zeatin, 1.0 mg/L NAA, 400 mg/L cefotaxime, 9 mg/L hygromycin, and 0.8% [*w*/*v*] agar). After 2–3 weeks of culture in the dark, leaf disks that generated calli were transferred to the screening medium (containing 2.0 mg/L zeatin, 0.1 mg/L NAA, 400 mg/L cefotaxime, 9 mg/L hygromycin, and 0.8% [*w*/*v*] agar). Regenerated shoots were transferred to the rooting medium (containing 0.1 mg/L NAA, 400 mg/L cefotaxime, 9 mg/L hygromycin, and 0.8% [*w*/*v*] agar). Finally, transgenic plants were selected with 9 mg/L hygromycin. Rooted plantlets were acclimatized in pots and then grown in a greenhouse. Transgenic positive plants were detected by PCR with gene-specific primers for the HPT gene (forward: 5′-ATCGGACGATTGCGTCGCATC-3′, reverse: 5′-GTGTCACGTTGCAAGACCTG-3′).

### 4.8. Transformation of A. thaliana

The genomic DNA fragment that contained the 1.5 kb upstream sequence of *PtoMYB055* was amplified with primers (forward: 5′-TGTACATATGATTAGAATAATGG-3′, reverse: 5′-TAGTCAGCAAATATATCCAATGATGC-3′). The PCR-amplified genomic DNA fragment was cloned into the plant binary vector pCXGUS-P [43]. We transformed the resulting vector PtoMYB055:GUS into wild-type plants using the floral dip method [47]. Transformants were selected on MS plates (containing 30 mg/mL hygromycin and 50 mg/mL carbenicillin). The first generation of 6-week-old transgenic plants was tested for expression of the GUS reporter gene [48].

### 4.9. Histochemical Staining of Lignin

Poplar stems from wild-type and transgenic plants were sectioned by hand at the fifth internode. The microsections were stained for 15 s with 1.0% (*w*/*v*) phloroglucinol after dissociation for 1 min by 40% (*v*/*v*) HCl and then observed under a microscope.

### 4.10. Determination of Lignin Autofluorescence

Poplar stems from wild-type and transgenic plants were sectioned by hand at the fifth internode. The stem cross-sections were dissected transversely with an Ultra-Thin Semiautomatic Microtome (FINESSE 325; Thermo Fisher Scientific, Waltham, MA, USA). Wood samples were observed under a confocal laser microscope (Leica, Wetzlar, Germany) following the manual’s recommendations.

### 4.11. Determination of Lignin Content

We used a modified Klason lignin method to measure the lignin content of poplar stems [49]. Briefly, freeze-dried poplar was ground with a mill to pass through a 40-mesh screen. The ground poplar (1 g) was then Soxhlet-extracted with 100 mL acetone for 8 h to remove extractable components. The total weight of the extractable components was determined gravimetrically by rotary evaporation. The extracted lignocellulosic material was dried to remove the solvent, and sugar and lignin were analyzed as follows. Three biological replicates were performed. The 0.2 g sample extracted from the poplar was weighed and transferred to a 15 mL reaction vial in an ice bath. Then, we added a 3 mL aliquot of 72% (*w*/*w*) H_2_SO_4_ to the sample and mixed it thoroughly for 60 s. Next, we put a test tube into a water bath immediately at 20 °C and mixed it for 1 min every 10 min. The contents of the test tube was transferred to a 125 mL serum bottle after 2 h of hydrolysis. We used 112 mL nanopure H_2_O to wash away all residue. Serum bottles, which contained 115 mL H_2_SO_4_ at 4% (*w*/*w*) plus poplar, were sealed with septa and autoclaved at 121 °C for 60 min. The sample were left at room temperature. The hydrolysates were vacuum-filtered through reweighed sintered-glass crucibles of medium coarseness, washed with 200 mL warm (approximately 50 °C) nanopure H_2_O to remove residual acid and sugars, and dried overnight at 105 °C. Finally, the dry crucibles were weighed to determine Klason (acid-insoluble) lignin gravimetrically.

### 4.12. Statistical Analyses

We used Student’s *t* test (http://www.graphpad.com/quickcalcs/ttest1.cfm) to analyze all data. Quantitative differences between the two groups in all experiments were statistically significant (*p* < 0.05).

### 4.13. GenBank Accession Numbers of Genes from Different Species

The accession numbers of the PtoMYB055in the GenBank database is KJ568513.1.

Other GenBank accession numbers for genes involved in this study are as follows: PtrCCOAOMT1 (EU603307.1), PtrCCR2 (EU603310.1), PtrCOMT2(EU603317.1), PtrC3H3 (EU603301.1), PtrPAL4 (EU603322.1),PtrC4H2 (EU603302.1), PtrCAD1 (EU603306.1), Ptr4CL5 (EU603299.1), PtrF5H2 (EU603311.1), PtrCesA2B (JX552008.1), PtrCesA3A (JX552264.1), PtrGT43B (JF518935.1), AtMYB20 (NC_003070.9), AtMYB43 (NC_003076.8), AtMYB46 (NM_121290), AtMYB58 (NC_003070.9), AtMYB61 (NC_003070.9), AtMYB83 (NM_111685), AtMYB85 (NC_003075.7), PtMYB1 (EU482902.1), PtMYB4 (AY356371), HvMYB1 (X70877.1), GhMYB1 (L04497.1), GhMYB6 (AF034134.1),EgMYB1 (AJ576024.1), ZmMYB31 (AM156906.1), ZmMYB42 (AM156908.1), OsMYB14 (Y11351.1), PtrMYB216 (XP_002319449.1), and OsMYB55 (Os05g0553400).

## Figures and Tables

**Table 1:** The lignin content in 35S-PtoMYB055-overexpressing poplar and the wild type.

Lines	Lignin (mg/100 mg)
Wild type	22.19 ± 1.24
35S:PtoMYB055 Line 1	24.47 ± 0.87
35S:PtoMYB055 Line 8	27.38 ± 2.15

## Supplementary Materials

Supplementary materials can be found at https://www.mdpi.com/1422-0067/21/14/4857/s1.

## Abbreviations

4CL	4-coumarate: CoA ligase
C4H	Cinnamate-4-hydroxylase
CCR	Cinnamoyl-CoA reductase
F5H	Coniferaldehyde 5-hydroxylase
CCoAOMT	Caffeoyl-coenzyme A 3-O-methyltransferase
NAC	NAM, ATAF1/2, and CUC2
RT-qPCR	Quantitative reverse transcription polymerase chain reaction
GUS	*β*-glucuronidase
CDS	Coding sequences
HCl	Phloroglucinol-hydrochloric acid
GFP	Green fluorescent protein
