# Supplementary material for: Identification and Functional Characterization of PtoMYB055 Involved in the Regulation of the Lignin Biosynthesis Pathway in Populus tomentosa
Source: Int J Mol Sci. 2020 Jul 9;21(14):4857. doi: 10.3390/ijms21144857 (PMC7402297; doi:10.3390/ijms21144857)
Supplement: Supplementary file 1 [file ijms-21-04857-s001.pdf]

Supplemental Table S1. Primers used in this study

Name	Forward Primer sequence	Reverse primer sequence
**Primers for transgenic plant detection**		
PtrMYB055	5´-CTTCGAATTTTCCCACATTTCTAAC-3´	5´-TCAAACTGATCCCCGGTCATC-3´
HPT	5′-CTTCTACACAGCCATCGGTCCAGA-3′	5′-GATGTAGGAGGGCGTGGATATGTC-3′
**Primers for Yeast-Hybrid**		
PtrMYB055-AD/BD	5'- GGATCCATGGGCAGATCTCCTTGTTGTG-3'	5'- GAATTCTCAAGCATCCAAAGGTCTGTAAAAT-3'
**Primers for Subcellular Localization**		
Sub- PtrMYB055	5´- GAATTCCATGGGCAGATCTCCTTGTTGTG -3´	5´- GAATTCTCAAGCATCCAAAGGTCTGTAAAAT -3´
**Primers for GUS activity assay (Promoters)**		
ProPtrMYB055	5´- GAATTCCAATCGTGGAAGGTAGGGGAAG -3´	5´- GAATTCAGAGGAGGGGATGGTGGTTATG -3´
**Primers for qRT–PCR**		
qPtrMYB055	5´-GATGTGGCAAAAGTTGTAGACTGAG-3´	5´-CTGGCAGCCTAGCAGCAATG-3´
qPtr18S	5´-GGCATGGAAGGTGATGCAGATC-3´	5´-CTGTGTCAAACAAGAACTTGTCC-3´
qPtrPAL4	5´-CCTACATTGACGATCCTTGCAG-3´	5´-GACCTGCATTCCTTGATCCTG-3´
qPtr4CL5	5´-CATCCGAGGTGATCAGATCATG-3´	5´-CACAGCAGCATCAGATATCC-3´
qPtrCHS	5'-TCACTGTTGAGACTGTGGTG-3'	5'-CACTCCTTATTGGTGCTCTC-3'

qPtrCHI	5'-TGTGCTAGAGTCAATGATTGG-3'	5'-GGAAAAGCTTGAGCCTGAAAT-3'
qPtrF3H	5'-TGGTCTGACTTTACAACGTGC-3'	5'-GACAACTCACACGGCATTGC-3'
qPtrDFR1	5'-GACAACTCACACGGCATTGC-3'	5'-TGCGATTGGCATTCGTCTAG-3'
qPtrANS1	5'-TGCGATTGGCATTCGTCTAG-3'	5'-GCAAGTGCATCGGAATGATC-3'
qPtrANR1	5'-CAGAGAAGCTCATCAGTGAG-3'	5'-AGACGCTAGATTGCTTCAGC-3'
qPtrLAR3	5'-CCTCCAAGCAATCTGCTAAGCACT-3'	5'-CCTCCAAGCAATCTGCTAAGCACT-3'
qPtrF5H2	5´-GAGTCCAGCAAGAGCTCGCAG-3´	5´-GCATAAGCATTGATCATCAC-3´
qPtrHCT1	5´-ATCAGCATGTAAGGCACGCGG-3´	5´-TGCCAAAGTAACCAGGTGGAAGCGT-3´
qPtrCCOAOMT1	5´-CAAGAGGTTGATTGAGCTTG-3´	5´-GGTCAGCAGCAAGTGCCTTG-3´
qPtrCCR2	5´-CTGTTCAAGCTTATGTGCATG-3´	5´-GTGGAGAACGCTCTCAGAGC-3´
qPtrCOMT2	5´-CATGAAGTGGATATGCCATG-3´	5´-GTTGAATGCACAGCACATTAC-3´
